# World Organization of National Colleges, Academies and Academic Associations of General Practitioners and Family Physicians (WONCA) Europe position paper on the use of point-of-care ultrasound (POCUS) in primary care

**DOI:** 10.1017/S1463423624000112

**Published:** 2024-04-23

**Authors:** Aaron Poppleton, Sonia Tsukagoshi, Shlomo Vinker, Francois Heritier, Paul Frappé, Fabian Dupont, Peter Sigmund, Mihai Iacob, Josep Vilaseca, Mehmet Ungan, Camilla Aakjær Andersen, Thomas Frese, David Halata

**Affiliations:** 1 School of Medicine, Keele University, Newcastle, UK; 2 European Young Family Doctors’ Movement, London, UK; 3 Tel Aviv University, Tel Aviv-Yafo, Israel; 4 Unisanté, Université de Lausanne, Lausanne, Switzerland; 5 Université Jean Monnet, Saint-Etienne, France; 6 Saarland University, Homburg, Germany; 7 Steirischen Akademie für Allgemeinmedizin, Graz, Austria; 8 Victor Babes University of Medicine and Pharmacy, Timișoara, Romania; 9 University of Barcelona, Barcelona, Spain; 10 School of Medicine, Ankara University, Ankara, Turkey; 11 Center for General Practice, Aalborg University, Aalborg, Denmark; 12 University Halle-Wittenberg, Halle (Saale), Germany; 13 European General Practice Research Network, Halle-Wittenberg, Germany; 14 POCUS iGP, Hošťálková, Czechia

**Keywords:** Medicine, point of care, position statement, primary care, family, ultrasound

## Statement

Point-of-care ultrasound (POCUS) has been introduced across a number of medical specialities, with emerging research showing promising results. We anticipate that POCUS will have an increasingly important place for specific indications within primary care over the coming years, supporting general practitioners to meet the health needs of their patient populations. We recommend that all general practitioners receive training in POCUS that is tailored to the needs of their healthcare context. This training should be delivered during general practitioners’ residency and continuing medical education programmes. Where evidence supports the use of POCUS in diagnosis, qualified general practitioners should be appropriately financed for its use in clinics, house calls and community healthcare. We support ongoing efforts to gather evidence for best practice use of POCUS and to explore the long-term effects of POCUS use on diagnosis within primary care.

## Background

POCUS is defined as ‘ultrasonography brought to the patient and performed by the provider in real time’ (Díaz-Gómez *et al.*, 2021). It is designed to answer a specific clinical question or to perform a specific procedural aim and is not a replacement for a formal ultrasound examination or screening (Andersen *et al*., 2019a; Díaz-Gómez et al., 2021). POCUS has been shown to be useful to rule in or rule out medical emergencies, to diagnose conditions of low to moderate complexity and to monitor acute and chronic illnesses independent of hospital infrastructures (AAFP, 2016; Andersen *et al*., 2019a; Colli *et al*., 2015; Genc *et al*., 2016; Myklestul *et al*., 2020; Sorensen and Hunskaar, 2019). Effective use of POCUS has been demonstrated in numerous clinical specialities for a wide range of indications, including those relating to: internal organs, such as the heart, lungs, and kidneys; musculoskeletal, soft tissue and vascular conditions; and pregnancy (AAFP, 2016; Andersen *et al*., 2020; Díaz-Gómez *et al*., 2021; Dietrich *et al*., 2017; Løkkegaard *et al*., 2020; Rodríguez-Contreras *et al*., 2022; Sorensen and Hunskaar, 2019). Exposure to and popularity of POCUS during undergraduate medical training has increased over the past decade (Dinh *et al*., 2016; Touhami *et al*., 2020).

Use of POCUS has been increasing in primary care (Myklestul *et al*., 2020; Touhami *et al*., 2020), with a strong interest among residents in family medicine to incorporate POCUS training into the family medicine curriculum (Peng *et al*., 2019; Andersen, *et al*., 2021b). Indications for POCUS vary between countries, shaped by the requirements of local health systems, the scope of primary care and training of general practitioners. Benefits of POCUS within primary care include its portability, ease of operation, high acceptability amongst patients and high user satisfaction amongst both patients and doctors (Andersen *et al*., 2019b; Andersen *et al*., 2021a; Iacob *et al*., 2016). POCUS can increase doctor confidence and studies suggest it can increase accuracy in diagnosis (Leidi *et al*., 2022). POCUS therefore has the potential to improve patient outcomes through a rapid initiation of effective treatment and a reduction in referrals to secondary care for investigations, specialist clinics and hospitalisation.(Colli *et al*., 2015; Andersen *et al*., 2020). POCUS has the potential to reduce health inequalities and empower general practitioners who work in rural, remote, under-resourced or underserved settings (Lo *et al*., 2022; Kornelsen *et al*., 2023; Tanael, 2021). We are supportive of further structured exploration and research in this area.

However, the use of POCUS in primary care is not without limitations. As with other physical examinations (eg, pulmonary auscultation and thyroid palpation), accuracy of POCUS is user-dependent (Díaz-Gómez *et al.,* 2021; Dietrich *et al*., 2017; Diprose *et al*., 2017). Compared with auscultation/clinical examination alone, focused use of POCUS has the potential to ensure higher levels of diagnostic accuracy and reduce risk of harm (Diprose *et al.*, 2017). Without adequate training and continuous utilisation, POCUS can lead to false reassurance, underdiagnosis, misdiagnosis, overdiagnosis and overtreatment (Andersen *et al*., 2019a. Leidi *et al*., 2020). Training should be stepwise and ongoing, including adequate coverage of anatomy and physiology, procedural techniques and communication skills including standardised reporting of clinical findings, and the impact of findings on medical decision-making in primary care (AAFP, 2016; Andersen *et al*, 2021b; Andersen, *et al*., 2022; Homar *et al*., 2020). Maintaining competency will be an important aspect of ongoing use of POCUS within a generalist speciality (EFUMB, 2006). More research is required to identify best practice in training, methods of assessment and quality improvement, including avoidance of overdiagnosis, within the context of primary care.

Medicolegal considerations vary across countries and frequently change. This will require providers and institutions to understand local regulatory requirements and legal frameworks to mitigate the potential risks of POCUS. Even, the stethoscope, a tool routinely used by physicians for over 200 years, has its limitations and failings (Arts *et al*., 2020). Reviews of POCUS-associated litigation within secondary care have not identified cases relating to the use of POCUS, but rather to the lack of POCUS use when the technology was available (Blaivas and Pawl, 2012; Conlon *et al*., 2022; Reaume *et al*., 2021). Assessing medicolegal risk is a preventative process to avoid harm, whether to the patient, provider or institution. Efforts must be made to gather evidence for guidelines on appropriate (and inappropriate) use of POCUS within primary care, in addition to the long-term impact on patient prognosis. We anticipate that specific regulatory frameworks for POCUS in General Practice are likely to evolve with an increased emphasis on quality and safety. We support the development of licensure and availability of General Practitioners to undertake POCUS in countries where this is not currently available.

## Conclusion

POCUS is an accessible and promising medical tool capable of increasing diagnostic value and accuracy within primary care. It has the potential to reduce healthcare costs, patient travel, waiting times, and need for referral to secondary care services. It does however have potential risks of underdiagnosis, misdiagnosis, overdiagnosis and overtreatment. We recommend that all general practitioners receive tailored curriculum-based training in POCUS during residency and continuing medical education programmes, with adequate financial provision to undertake POCUS within primary care. We suggest that open dialogue and partnership with providers, administrators and regulatory agencies experienced in POCUS will enable development of strategies to improve availability, provider performance, patient outcomes and minimisation of risk.

### Addendum: Application of the WONCA Europe position statement

The World Organization of National Colleges, Academies and Academic Associations of General Practitioners and Family Physicians (WONCA) Europe represents 47 member organisations consisting of more than 120,000 general practitioners in Europe. The position statement is a general endorsement of POCUS within family medicine within the European region, irrespective of clinic size, staff composition, licensure, governance procedures and financing of services. Authors of this position statement represent this variation, including large multidisciplinary practices, academic/training settings, urban/suburban/rural localities and single-handed practices. Ultrasound is a complex and user-dependent investigation. Appropriate training and continuing medical education is required to maintain competency, meet local population health needs and fulfil national regulatory requirements. We encourage dissemination and mutual learning from effective training approaches and funding models within European localities to support effective use of POCUS in family medicine.
